# Use of Oral Anticoagulation and Diabetes Do Not Inhibit the Angiogenic Potential of Hypoxia Preconditioned Blood-Derived Secretomes

**DOI:** 10.3390/biomedicines8080283

**Published:** 2020-08-11

**Authors:** Philipp Moog, Maryna Jensch, Jessica Hughes, Burak Salgin, Ulf Dornseifer, Hans-Günther Machens, Arndt F. Schilling, Ektoras Hadjipanayi

**Affiliations:** 1 Experimental Plastic Surgery, Clinic for Plastic, Reconstructive and Hand Surgery, Klinikum Rechts der Isar, Technische Universität München, D-81675 Munich, Germany; philippmoog@web.de (P.M.); marinajensch@gmx.de (M.J.); Jessica.Hughes@gmx.de (J.H.); ulf.dornseifer@isarklinikum.de (U.D.); e.hadjipanayi@gmail.com (E.H.); 2 Centre for Neuroscience, Surgery and Trauma, Blizard Institute, Barts and The London School of Medicine and Dentistry, Queen Mary University of London, London E1 4NS, UK; b.salgin@qmul.ac.uk; 3 Department of Plastic, Reconstructive and Aesthetic Surgery, Isar Klinikum, D-80331 Munich, Germany; 4 Department of Trauma Surgery, Orthopedics and Plastic Surgery, Universitätsmedizin Göttingen, D-37075 Göttingen, Germany; arndt.schilling@med.uni-goettingen.de

**Keywords:** acetylsalicylic acid, angiogenesis, blood-derived therapy, COX-1, clopidogrel, drug anticoagulation, hypoxia, hypoxia preconditioned plasma, hypoxia preconditioned serum, NOACs, peripheral blood cells, oral anticoagulation, VEGF

## Abstract

Patients suffering from tissue ischemia, who would greatly benefit from angiogenesis-promoting therapies such as hypoxia preconditioned blood-derived secretomes commonly receive oral anticoagulation (OA) and/or have diabetes mellitus (DM). In this study, we investigated the effect of OA administration on the in vitro angiogenic potential of hypoxia preconditioned plasma (HPP) and serum (HPS), prepared from nondiabetic/diabetic subjects who did not receive OA (*n* = 5) or were treated with acetylsalicylic acid (ASA, *n* = 8), ASA + clopidogrel (*n* = 10), or nonvitamin K antagonist oral anticoagulants (*n* = 7) for longer than six months. The effect of DM was differentially assessed by comparing HPP/HPS obtained from nondiabetic (*n* = 8) and diabetic (*n* = 16) subjects who had not received OA in the past six months. The concentration of key proangiogenic (vascular endothelial growth factor or VEGF) and antiangiogenic (thrombospondin-1 or TSP-1 and platelet factor-4 or PF-4) protein factors in HPP/HPS was analyzed via ELISA, while their ability to induce microvessel formations was examined in endothelial cell cultures. We found that OA use significantly reduced VEGF levels in HPP, but not HPS, compared to non-OA controls. While HPP and HPS TSP-1 levels remained largely unchanged as a result of OA usage, HPS PF-4 levels were significantly reduced in samples obtained from OA-treated subjects. Neither OA administration nor DM appeared to significantly reduce the ability of HPP or HPS to induce microvessel formations in vitro. These findings indicate that OA administration does not limit the angiogenic potential of hypoxia preconditioned blood-derived secretomes, and therefore, it does not prohibit the application of these therapies for supporting tissue vascularization and wound healing in healthy or diabetic subjects.

## 1. Introduction

Wounds normally heal via a set of complex and interactive phases that include hemostasis, inflammation, proliferation, and remodeling [1,2,3,4]. This well-orchestrated wound-healing process can be impaired by various local and systemic factors, causing further complications and a lower quality of life for patients [4]. A chronic wound results when the healing program, which becomes activated following tissue injury, does not orderly progress through the aforementioned stages and is thus unable to complete the sequence of biological events that physiologically lead to angiogenic induction [4,5]. This failure to stimulate vascularization, and supply traumatized tissue with oxygen and nutrients, is what forces the development of chronic wounds, which clinically are directly related to poor tissue perfusion [6]. Chronic tissue ischemia, frequently manifested in the form of atherosclerotic or diabetes-induced wound-healing disorders, is a leading disease in the Western world and is associated with high morbidity and mortality [7,8], while its prevalence is steadily increasing [2,4,7,9].

Atherosclerosis is a progressive inflammatory disease leading to atherosclerotic plaques that cause narrowing of the arterial lumen [7,10,11,12,13]. Restriction of the vessel lumen and the presence of atherosclerotic plaques are linked to an increased risk of cardiovascular events such as myocardial infarction, stroke, and peripheral ischemia [7,10,11,13,14]. Among those suffering from peripheral ischemia, 20% to 70% have chronic leg ulcers [15]. Without timely, appropriate interventions, progressive atherosclerotic plaque-related ischemia increases the one-year risk of lower limb wound deterioration by 35% [15,16]. Similar to atherosclerosis, the dysregulation of wound healing in diabetes is mainly characterized by a series of micro- and macrovascular changes, chronic inflammation, disruption of angiogenic processes, and an imbalance in extracellular matrix regulation [4,8,17,18,19]. The persistent hyperglycemic state in diabetic patients results in endothelial dysfunction and smooth muscle abnormalities, followed by vasoconstriction due to the reduction of vasodilators [20]. Moreover, hyperglycemia correlates with stiffer blood vessels, which causes alterations in blood flow and, consequently, reduced tissue oxygenation [8,18,21]. Vascular pathology also contributes to reduced leukocyte migration into the wound, which becomes more vulnerable to infections [18,22]. Importantly, diabetic wounds are negatively impacted by insufficient angiogenesis (new vessel formation), as they show decreased vascularity and capillary density [23,24].

While the etiology of chronic, nonhealing wounds is multifaceted and specific pathology-dependent, the progression to a nonhealing phenotype is consistently closely linked to poor tissue perfusion [23,25,26], which, consequently, leads to tissue ischemia. As a means of normalizing the blood flow profile, oral anticoagulation (OA) is often necessary when treating patients suffering from atherosclerotic and/or diabetic microangiopathies [27], and it is commonly administered as an adjuvant therapy for vascular stenosis in the context of vascular interventions such as vascular dilation and stenting [28]. For example, in symptomatic peripheral arterial disease, single antiplatelet therapy with acetylsalicylic acid (ASA) or clopidogrel is indicated [29]. In patients undergoing percutaneous peripheral interventions for significantly stenosed blood vessels, at least four weeks of dual antiplatelet therapy with ASA and clopidogrel is recommended after infrainguinal stent implantation, while stenting below-the-knee arteries is often followed by an even longer period of dual antiplatelet therapy [29]. Often, however, these treatments are unsatisfactory, and there are, so far, no established therapeutic options that can improve local tissue perfusion by actively supporting angiogenesis in the wound microenvironment [8,30,31,32,33,34,35].

Our previous work provided evidence that hypoxia preconditioned blood-derived secretomes could constitute a new generation of autologous and bioactive topically applied/injectable compositions that can supply the necessary biochemical signals for initiating and supporting dermal fibroblast proliferation/migration and angiogenesis in injured tissue, thus driving wound healing to completion [34,35,36,37]. Our latest data also suggest that, beyond promoting angiogenesis, hypoxia preconditioned secretomes also have the ability to induce lymphangiogenesis, another important biological process in wound healing (work in progress). These angiogenic growth factor mixtures can be obtained through extracorporeal wound simulation (EWS), a method that employees peripheral blood conditioning outside the body [34,37,38]. Conditioning peripheral blood cells (PBCs) under the very same conditions that are normally encountered within a healing wound, i.e., physiological temperature and hypoxia, offers a means of optimizing the angiogenic potential of hypoxia preconditioned secretomes, i.e., hypoxia preconditioned plasma (HPP) and hypoxia preconditioned serum (HPS), which can be readily and selectively prepared in the clinical setting by adjusting blood coagulation prior to hypoxic conditioning [34,37,38,39]. HPP and HPS constitute phase-specific growth factor secretomes, since each corresponds to a different phase of the wound-healing cascade—specifically, HPP being more closely correlated with the hypoxia-induced upregulation of proangiogenic growth factors that are produced by leukocytes, while HPS comprising a combination of coagulation-mediated platelet-derived protein factors and hypoxia-induced signaling [34,38,39]. The differences in proteomic composition that result from this selective adjustment of blood coagulation have already been extensively characterized by our group, showing that, while HPP has a comparable concentration of the proangiogenic factor vascular endothelial growth factor (VEGF) to HPS, it has a lower concentration of the platelet-derived angiogenic inhibitors thrombospondin-1 (TSP-1) and platelet factor-4 (PF-4) [37,39]. Despite these differences in protein factor concentrations, both HPP and HPS appear to induce similar levels of microvessel formation and sprouting in vitro [39].

As previously mentioned, many patients requiring angiogenesis-promoting therapies for central/peripheral vasculopathy and chronic wounds, who would greatly benefit from new generation bioactive treatments such as hypoxia preconditioned blood-derived secretomes, routinely receive OA [27,40,41]. A better understanding of the effects that OA administration may have on the angiogenic potential of blood-derived secretomes is therefore a key prerequisite for advancing their clinical utility. Moreover, the high frequency of the coprevalence of atherosclerotic disease and diabetes in this target patient group necessitates a differential investigation of the effects that OA administration and diabetic pathology may have on peripheral blood cell angiogenic functions. In the current study, we aimed to characterize the proteomic composition of HPP and HPS derived from peripheral blood that was obtained from nondiabetic and diabetic subjects receiving OA medications, specifically acetylsalicylic acid (ASA), a combination of ASA and clopidogrel, and nonvitamin K antagonist oral anticoagulants (NOACs), in terms of key proangiogenic (VEGF) and antiangiogenic (PF-4 and TSP-1) growth factors. Furthermore, an attempt was made to differentially analyze the influence of OA administration and diabetic pathology on the secretomes’ ability to induce microvessel formations in vitro. Beyond providing useful insights into the clinical utility of these potentially therapeutic products, this work could also enhance our scientific understanding of the interactive roles that coagulation-mediated and hypoxia-induced protein factor signaling may have in wound angiogenesis.

## 2. Experimental Section

### 2.1. Study Collective

All blood donors provided written informed consent as directed by the ethics committee of the Technical University Munich, Germany, which approved this study (File Nr.: 497/16S; Amendment; date of approval: 19 of August 2017). The use of oral anticoagulation (OA) was due to previous comorbidities (2 subjects: strokes, 18 subjects: cardiac disease, 4 subjects: peripheral arterial diseases, and 5 subjects: thromboembolic events; note: some subjects received OA due to multiple comorbidities, which led to 29 comorbidities in 25 subjects). Subjects were recruited in our clinic in 2018–2019, while inclusion of subjects in the study was done on a voluntary basis. All subjects were screened for drug-mediated blood anticoagulation and diabetes mellitus and exclusion criteria. We excluded subjects who were under a non-consistent blood anticoagulation regime in the past 6 months or had a diagnosis for diabetic disease for less than one year, as well as subjects who suffered from mental disorders (e.g., dementia and psychosis). Further comorbidities such as heart disease were not considered as exclusion criteria. Smokers were defined as those who had smoked more than one cigarette in the past three months.

For OA experiments, we included and evaluated 25 subjects with an average age of 70.64 ± 7.22 years (Table 1A). Subjects were divided into three groups, according to the type of OA administered. We tested the effect of three of the most widely used oral blood anticoagulants: Group 1, acetylsalicylic acid (ASA) (100 mg/day), group 2, a combination of ASA + clopidogrel (Plavix) (100 mg + 75 mg/day), and group 3, nonvitamin K antagonist oral anticoagulants/factor-Xa-inhibitors (NOACs) (e.g., Apixaban and Rivaroxaban; dose was dependent on pathology and drug type). All subjects were under a consistent blood anticoagulation regime in the past 6 months. Seven subjects who took oral anticoagulation (OA) suffered from type 2 diabetes (T2D) (Table 1A).

To assess the effect of diabetes mellitus (DM) independently of OA use, we included and evaluated 16 diabetic subjects with an average age of 52.09 ± 18.86 years, of which 6 subjects had a clinical diagnosis of type 1 diabetes (T1D) and 10 subjects of type 2 diabetes (T2D) for longer than one year prior to the initiation of the study (Table 1B). None of the 16 DM subjects had received oral anticoagulation in the past 6 months, but all took medication to control blood sugar. Blood glucose was determined immediately after taking blood for HPP/HPS preparation. The timing of drawing blood was independent of prior food intake.

Control subjects were young healthy adults who were not taking any medication, including oral anticoagulation (OA), and without any known comorbidities. A total of 13 subjects were included: 5 of them serving as a control group for examining OA administration and 8 as a control group for examining the effects of DM. Table 1 provides information on the patient demographics.

### 2.2. Preparation of Blood Plasma/Serum and Hypoxia Preconditioned Plasma (HPP)/Serum (HPS) Samples

**Figure 1:**
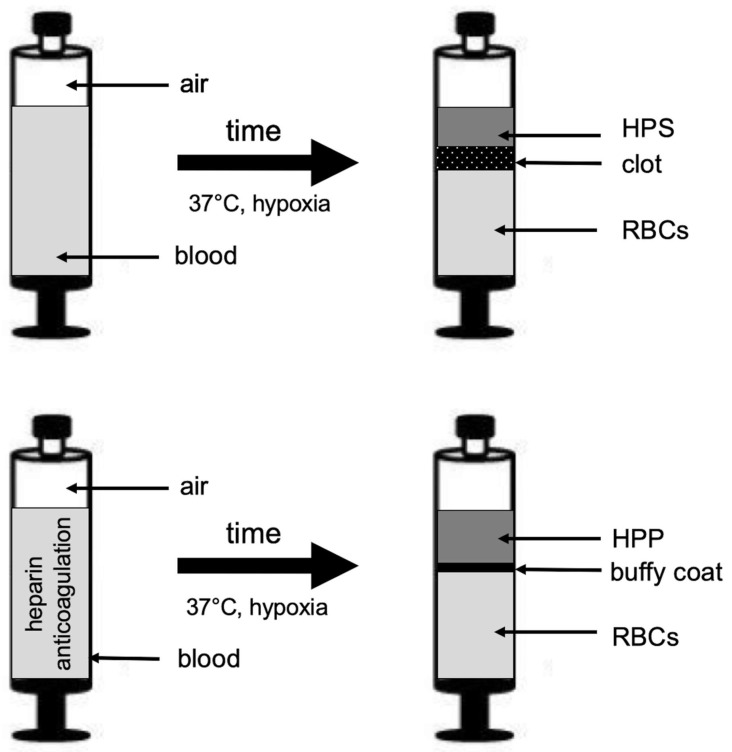
Method of preparation of hypoxia preconditioned plasma and serum (HPP and HPS). Peripheral venous blood obtained from subjects that received no oral anticoagulation (OA) or were on treatment with oral anticoagulants (acetylsalicylic acid (ASA), ASA + clopidogrel, or nonvitamin K antagonist oral anticoagulants (NOACs)) was allowed to clot (upper schematic) or additionally anticoagulated with heparin (bottom schematic) for HPS and HPP preparation, respectively. Peripheral blood cells (in clot or buffy coat) were conditioned under pericellular (local) hypoxia (~1% O_2_) and physiological temperature (37 °C) for 4 days. Sedimentation passively separated growth factor-rich HPS and HPP from clot and buffy coat, respectively, while red blood cells (RBCs) collected at the bottom of the chamber.

Peripheral venous blood (10 mL) was collected from all study participants (*n* = 54) into a 10-mL polypropylene syringe (Omnifix^®^, Braun AG, Melsungen, Germany) that contained no additive for normal serum and HPS preparation or was prefilled with 1-mL heparin (Medunasal^®^, Heparin 500 I.U. 5-mL ampoules, Sintetica^®^, Münster, Germany) for normal plasma and HPP preparation, under sterile and standardized conditions (Blood Collection Set 0.8 × 19 mm × 178 mm; Safety-Lok, CE 0050, BD Vacutainer, BD, NJ, USA). For normal plasma/serum preparation, following passive sedimentation for 60 min at room temperature (25 °C, no centrifugation) the blood was separated into three layers, from bottom to top: red blood cell component (RBCs), clot/buffy coat, and serum/plasma, so that the top layer (serum or plasma) could be filtered into a new syringe. For HPP/HPS preparation, following blood sampling a 0.2-µm pore filter was attached to the syringe (Sterifix^®^, CE 0123, Braun AG, Melsungen, Germany), and by pulling the plunger, 2 mL of air was drawn into the syringe through the filter (note that the 10-mL polypropylene syringe has a 13-mL capacity when pulled up to the stop). Subsequently, the filter was removed, and the capped syringe was placed upright in an incubator (37 °C/5% CO_2_) and incubated for 4 days (blood incubation time) without any prior centrifugation (Figure 1). Pericellular local hypoxia (~1% O_2_) was induced in situ through cell-mediated O_2_ consumption by controlling the blood volume per unit area (BVUA > 1 mL/cm^2^) and, consequently, the PBC seeding density in the blood container [34,42]. After the predefined incubation time, the blood was passively separated into three layers (Figure 1), from top to bottom: HPP/HPS, buffy coat/clot, and red blood cell (RBC) component, so that the top layer comprising hypoxia preconditioned plasma or serum could be filtered (0.2-µm pore filter, Sterifix^®^, Braun AG, Melsungen, Germany) into a new syringe, removing cells/cellular debris. 

### 2.3. Quantitative Analysis of VEGF, TPS-1, and PF-4 Concentrations in Blood-Derived Secretomes

Blood-derived secretomes (normal plasma/serum, HPP, and HPS) were sampled and analyzed by ELISA for VEGF, TSP-1, and PF-4 (R&D Systems, Inc., Minneapolis, MN, USA), according to the manufacturer’s instructions. Factor concentrations in hypoxia preconditioned blood-derived secretomes (HPP/HPS) were measured immediately after the predefined incubation period (4 days). At least one well was tested per subject per condition. 

### 2.4. Analysis of the Effect of Blood-Derived Secretomes on Microvessel Formation In Vitro

The angiogenic potential of blood-derived secretomes was tested in an in vitro angiogenesis assay by assessing their ability to induce microvessel formations in human umbilical vein endothelial cells (HUVECs, CellSystems, Troisdorf, Germany) seeded on factor-reduced Matrigel (BD, Heidelberg, Germany). HUVECs were seeded at a density of 10 × 10^3^/well, with 50 μL of test or control media added per well (μ-Slide angiogenesis, Ibidi, Gräfelfing, Germany), and cultured in a 5% CO_2_/37 °C incubator for 12 h. Cells were then stained with Calcein AM (PromoKine, Heidelberg, Germany), and endothelial cell tube formation was observed with fluorescence and phase contrast microscopy. Assessment of the extent of capillary-like network formation was carried out by counting the number of tubes and nodes (a node was defined as the point of intersection of two or more tubules). Hypoxia preconditioned blood-derived secretomes (HPP/HPS) were tested immediately after the predefined incubation period (4 days). Phosphate-buffered saline (PBS) medium and recombinant VEGF (90 ng/mL) were also tested as negative and positive controls, respectively. At least three wells were tested per sample per condition.

### 2.5. Statistical Analysis

Statistical analysis was carried out using a Student’s independent *t*-test, where a maximum of two groups was compared, or one-way ANOVA with Bonferroni adjustment, accompanied by post-hoc pairwise comparisons for analyses of more than two groups, using SPSS 14 software (version 14, IBM, Ehningen, Germany). The probability of a type-one error was set to 5% (α = 0.05), unless noted otherwise. For each experimental condition, blood-derived secretome samples from at least 5 subjects were tested, as noted in Table 1. Data are expressed as mean ± standard deviation.

## 3. Results

### 3.1. Effect of Oral Anticoagulation on Pro- (VEGF) and Anti-(TSP-1 and PF-4)Angiogenic Growth Factor Concentrations in Hypoxia Preconditioned Blood-Derived Secretomes 

**Figure 2:**
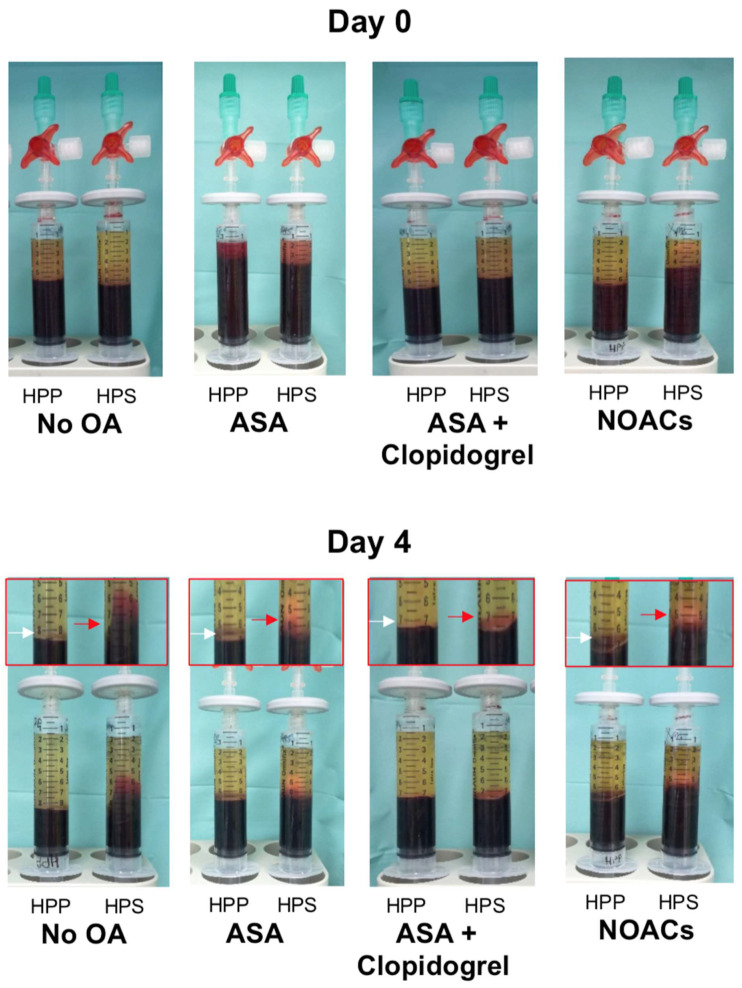
Representative images of hypoxia preconditioned plasma and serum (HPP and HPS) samples, obtained from subjects not receiving oral anticoagulation (OA) or subjects using acetylsalicylic acid (ASA), ASA + clopidogrel, or nonvitamin K antagonist oral anticoagulants (NOACs). Upper panel shows samples immediately after blood collection (day 0), while lower panel shows samples following 4 days of blood conditioning (sedimentation and hypoxic incubation). Enlarged image sections, indicated by red insets on day 4 images, show an enlarged view of the buffy coat layer (white arrow) and clot (red arrow) in 4-day incubated-HPP and -HPS samples, respectively.

The primary aim of this study was to examine whether the administration of oral anticoagulation (OA) influences the angiogenic properties of blood-derived hypoxia preconditioned secretomes. The first question we sought to answer was whether the use of OA prevents clotting in situ during the preparation of hypoxia preconditioned serum (HPS) from peripheral venous blood. As can be seen in Figure 2, clotting occurred normally in all HPS samples, after four days of blood incubation, regardless of the type of OA administered, although slightly smaller clots were present in HPS samples obtained from patients receiving a combination of ASA and clopidogrel.

**Figure 3:**
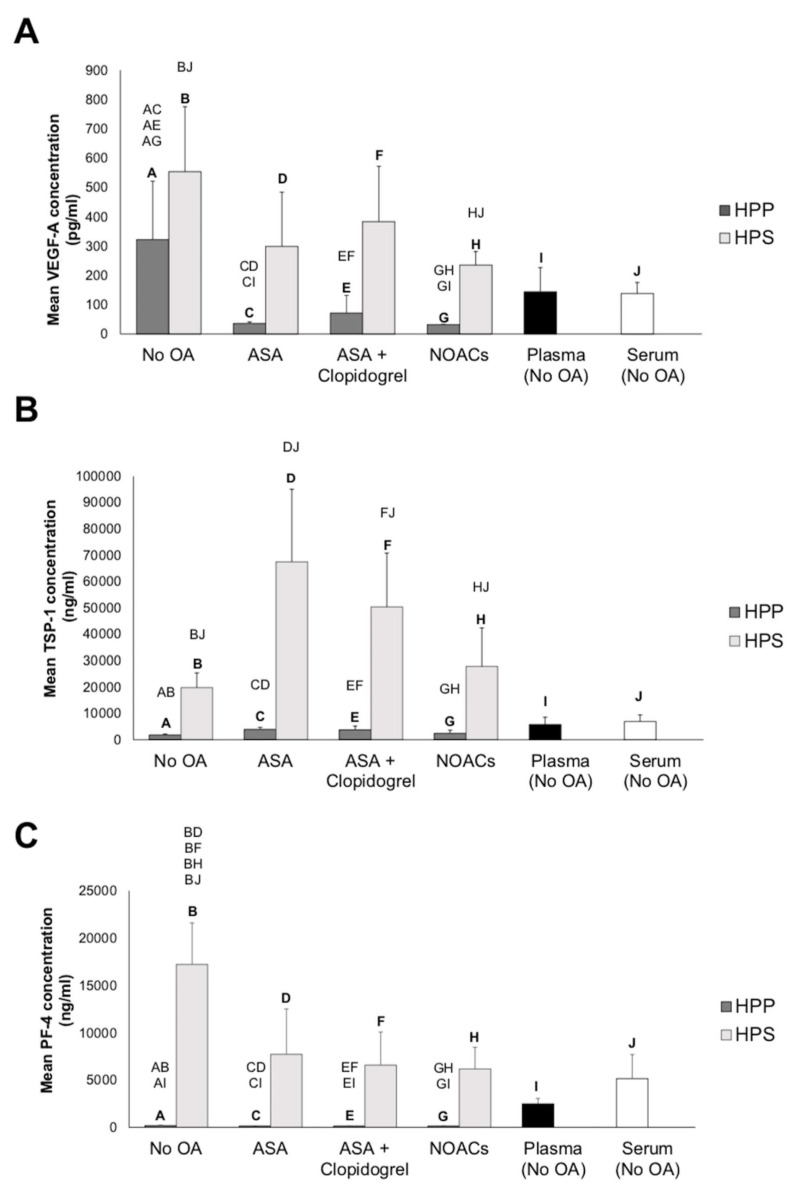
Quantitative analysis of pro- (vascular endothelial growth factor or VEGF) and antiangiogenic (thrombospondin-1 or TSP-1 and platelet factor-4 or PF-4) factor concentrations in blood-derived secretomes obtained with or without oral anticoagulation (OA). Plots showing the concentrations of (**A**) VEGF (pg/mL), (**B**) TSP-1 (ng/mL), and (**C**) PF-4 (ng/mL) in blood-derived secretomes: hypoxia preconditioned plasma (HPP), hypoxia preconditioned serum (HPS), normal plasma, and normal serum. HPP and HPS samples were obtained from subjects that received no oral anticoagulation (OA) or were on treatments with acetylsalicylic acid (ASA), ASA + clopidogrel, or nonvitamin K antagonist oral anticoagulants (NOACs). Capital letter pairs over plots indicate statistical comparisons of the corresponding data points. For all pair comparisons, *p* < 0.05, unless otherwise indicated. Error bars represent s.d.; number of subjects tested: no OA, *n* = 5; ASA, *n* = 8; ASA + clopidogrel, *n* = 10; NOACs, *n* = 7; normal plasma (no OA), *n* = 5; and normal serum (no OA), *n* = 5. At least one assay well was tested per subject per condition.

To establish a growth factor concentration baseline, we quantitatively analyzed via ELISA the concentration of key pro- and antiangiogenic protein factors (VEGF, TSP-1, and PF-4) in normal plasma and serum and compared them to their hypoxia-conditioned counterparts, obtained from healthy subjects that did not receive OA. As shown in Figure 3A, the concentration of the proangiogenic factor VEGF in hypoxia preconditioned plasma (HPP) and serum (HPS) showed a three-to-five-fold increase compared to its baseline level in fresh plasma (*p* > 0.05) and fresh serum (*p* < 0.05), respectively. There was no significant difference between HPP and HPS VEGF levels in these subjects (*p* > 0.05). In contrast, for subjects receiving OA (ASA, ASA + clopidogrel, or NOACs), HPP appeared to consistently have a significantly lower VEGF concentration than HPS (*p* < 0.05), regardless of the type of OA administered, but, also, a lower VEGF concentration compared to HPP obtained with no OA (*p* < 0.05), as well as fresh plasma, although this difference was only significant for HPP obtained from subjects receiving ASA or NOACs (*p* < 0.05).

Examination of the angiogenic inhibitor TSP-1 in these secretomes showed a significantly lower concentration in HPP compared to HPS (*p* < 0.05). This difference was present with or without OA administration (Figure 3B). Moreover, HPS obtained from all subjects had a significantly higher TSP-1 concentration compared to normal serum (*p* < 0.05). In contrast, the TSP-1 concentration of HPP obtained with or without OA appeared to be somewhat lower than that of normal plasma, although this difference was not significant (*p* > 0.05). 

Quantification of the PF-4 concentration yielded similar results to TSP-1, with HPP having a significantly lower concentration compared to HPS, regardless of whether blood had been obtained with or without OA intake (*p* < 0.05) (Figure 3C). In contrast to the TSP-1 concentration, however, the HPP PF-4 concentration was significantly below that of normal plasma for all conditions tested (*p* < 0.05). Furthermore, HPS obtained from subjects not receiving OA appeared to have a two-to-three-fold higher PF-4 concentration compared to HPS obtained with OA administration, as well as normal serum (*p* < 0.05). 

### 3.2. Quantitative Analysis of Blood-Derived Secretome Pro- (VEGF) and Anti-(TSP-1 and PF-4)Angiogenic Growth Factor Concentrations in Diabetic Subjects

**Figure 4:**
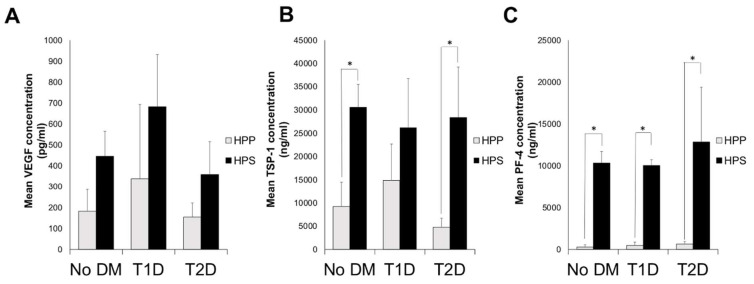
Comparative analysis of pro- (VEGF) and antiangiogenic (TSP-1 and PF-4) factor concentrations in hypoxia preconditioned blood-derived secretomes obtained from diabetic and nondiabetic subjects. Plot showing the concentrations of (**A**) VEGF (pg/mL), (**B**) TSP-1 (ng/mL), and (**C**) PF-4 (ng/mL) in HPP and HPS samples obtained from subjects suffering from type 1 (T1D) or type 2 (T2D) diabetes mellitus (DM) and nondiabetic subjects (no DM). For all pair comparisons, * *p* < 0.05, unless otherwise indicated. Error bars represent s.d.; number of subjects tested: no DM, *n* = 8; T1D, *n* = 6; and T2D, *n* = 10. At least one assay well was tested per subject per condition.

Oral anticoagulation is commonly prescribed to patients suffering from peripheral vascular pathology [43], which is commonly associated with diabetes mellitus [44]. Indeed, in our study collective, seven subjects receiving OA had concomitant type 2 diabetes (T2D) (see Table 1A). In order to assess whether the presence of diabetes exerted a bias in our analysis of the influence of OA administration on blood-derived secretome angiogenic composition (note; all subjects not receiving OA were non-diabetic), we examined the concentration of VEGF, TSP-1 and PF-4 in hypoxia preconditioned plasma and serum (HPP, HPS) samples, derived from non-diabetic and diabetic subjects who had not taken OA in the past 6 months. As shown in Figure 4A–C, no significant differences could be seen in the levels of these three protein factors between diabetic and non-diabetic subjects, in either HPP or HPS. Furthermore, the HPP/HPS concentration of these three factors was not significantly different in subjects suffering from type 1 or type 2 diabetes (Figure 4). Examination of the platelet-derived angiogenic inhibitors TSP-1 and PF-4 in these secretomes did show a significantly lower concentration in HPP compared to HPS (*p* < 0.05), in agreement with our previous data (Figure 3B,C), while this difference was present with or without T1D/T2D (Figure 4B,C), except for T1D subjects who had similar levels of TSP-1 in HPP and HPS. These results indicated that diabetes mellitus was likely not a confounding factor in the investigation of the effect of OA administration on HPP/HPS angiogenic potential.

### 3.3. Effects of Oral Anticoagulation on the Ability of Hypoxia Preconditioned Blood-Derived Secretomes to Induce Angiogenesis In Vitro

**Figure 5:**
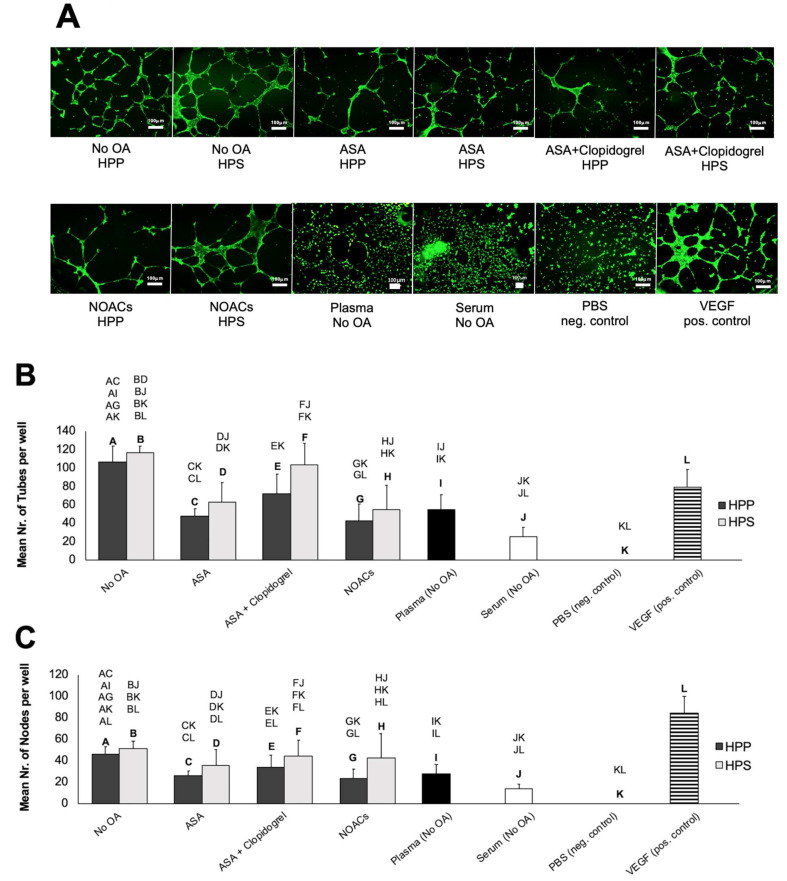
Effects of hypoxia preconditioned blood-derived secretomes obtained with or without oral anticoagulation (OA) on microvessel formations in human umbilical vein endothelial cell (HUVEC) cultures in vitro. (**A**) Panel showing representative images of the tube formation assay (12 h) carried out in the presence of blood-derived secretomes (HPP, HPS, normal plasma, and normal serum) obtained from subjects that did not receive oral anticoagulation (OA) or were treated with acetylsalicylic acid (ASA), ASA + clopidogrel, or nonvitamin K antagonist oral anticoagulants (NOACs) (Bars = 100 μm). (**B**) Plots showing the mean number of tubes and (**C**) nodes formed in HUVEC cultures that were incubated for 12 h with blood-derived secretomes. Capital letter pairs over plots indicate statistical comparisons of the corresponding data points. For all pair comparisons, *p* < 0.05, unless otherwise indicated. Error bars represent s.d.; number of subjects tested: no OA, *n* = 5; ASA, *n* = 8; ASA + clopidogrel, *n* = 10; NOACs, *n* = 7; normal plasma (no OA), *n* = 5; normal serum (no OA), *n* = 5; phosphate-buffered saline (PBS), *n* = 3; and VEGF, *n* = 3. Three samples were tested per subject per condition.

Following an analysis of key pro- and antiangiogenic protein factors in blood-derived secretomes obtained from subjects receiving OA, we moved on to investigate the effects of OA administration on the secretomes’ ability to induce microvessel formations in human umbilical vein endothelial cell (HUVEC) in vitro cultures. In subjects not receiving OA, HPP and HPS induced two-to-three times as many tubes and nodes (microvessel intersections) as fresh plasma and serum, respectively (*p* < 0.05) (Figure 5A–C). Additionally, HPS obtained from these subjects, but not HPP, appeared to perform somewhat better than pure recombinant VEGF, tested here as a positive control, in terms of the number of tubes formed (*p* < 0.05) (Figure 5B). In subjects receiving OA, however, only HPS appeared to be more angiogenic than fresh serum (*p* < 0.05), with HPP samples evoking a weaker angiogenic response that was comparable to that of normal plasma (Figure 5A–C). Notably, HPS obtained from ASA-treated subjects generated less tubes (although a similar number of nodes) compared to control HPS. Moreover, HPS obtained from subjects receiving OA failed to surpass the response produced by pure VEGF, while HPP obtained from ASA- and NOAC-treated subjects generated a significantly lower response than control HPP and positive control VEGF samples (*p* < 0.05) (Figure 5B,C). Importantly, no significant differences were observed in the number of tubes or nodes formed in cultures incubated with HPP or HPS, regardless of whether subjects were receiving OA or not (Figure 5A–C).

### 3.4. Influence of Diabetes Mellitus on the Ability of Hypoxia Preconditioned Blood-Derived Secretomes to Induce Angiogenesis In Vitro

**Figure 6:**
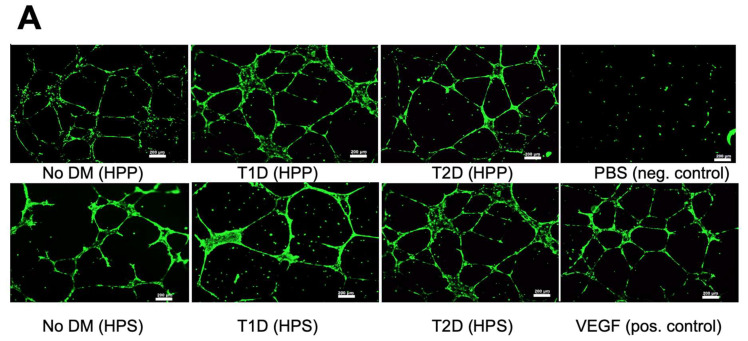

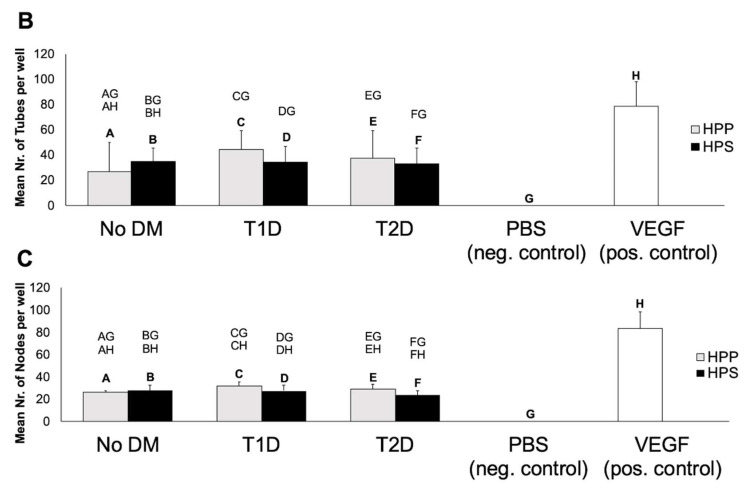
Effects of hypoxia preconditioned blood-derived secretomes obtained from diabetic subjects on microvessel formations in human umbilical vein endothelial cell (HUVEC) cultures in vitro. (**A**) Panel showing representative images of the tube formation assay (12 h), carried out in the presence of hypoxia preconditioned plasma and serum (HPP and HPS) obtained from subjects suffering from type 1 (T1D) or type 2 (T2D) diabetes, as well as nondiabetic subjects (no DM) (Bars = 200 μm). (**B**) Plots showing the mean number of tubes and (**C**) nodes formed in HUVEC cultures that were incubated for 12 h with HPP and HPS samples obtained from the above test conditions. Capital letter pairs over plots indicate statistical comparisons of the corresponding data points. For all pair comparisons, *p* < 0.05, unless otherwise indicated. Error bars represent s.d.; number of subjects tested: no DM, *n* = 8; T1D, *n* = 6; T2D, *n* = 10; PBS, *n* = 3; and VEGF, *n* = 3. Three samples were tested per subject per condition.

After having shown that the presence of diabetes does not significantly influence the concentration of key pro- and antiangiogenic factors in HPP/HPS, we proceeded by examining whether this translated into an equal ability of hypoxia preconditioned secretomes, obtained from diabetic and nondiabetic subjects who did not receive OA in the past six months, to induce microvessel formations in vitro. As shown in Figure 6A–C, no significant differences could be seen as a result of diabetic pathology in the mean number of tubes and nodes formed in human umbilical vein endothelial cell (HUVEC) cultures following 12-h incubation with either HPP or HPS. While all samples induced a stronger angiogenic response than the negative control (*p* < 0.05), all conditions underperformed pure VEGF (positive control) in terms of node formation (*p* < 0.05) (Figure 6B and Figure 5C). Furthermore, and in agreement with our ELISA results, HPP/HPS-induced microvessel formations did not significantly differ between T1D and T2D subjects (Figure 6A–C).

## 4. Discussion

Hypoxia preconditioned blood-derived secretomes represent a new development in the field of autologous growth factor therapies that could prove to be beneficial to many patients requiring angiogenesis-promoting treatments for central/peripheral ischemia and chronic/diabetic wounds [34,35,36,37,38,39]. These autologous growth factor preparations are produced through the extracorporeal conditioning of peripheral blood cells (PBCs) under wound-simulating conditions [34,38]. We previously described the use of hypoxia preconditioned plasma (HPP), i.e., plasma derived after conditioning anticoagulated blood under a physiological temperature (37 °C) and physiological hypoxia (1–10% O_2_) for four days (Figure 1) as a tool for stimulating microvessel formation and sprouting [34,39]. Similarly, conditioning blood in a nonanticoagulated state yields hypoxia preconditioned serum (HPS) (Figure 1), which (at least, in vitro) was found to be equally angiogenic as HPP [37,39]. In terms of the growth factor composition, while HPP comprises primarily PBC-derived hypoxia-induced signaling, HPS represents a more “complete” secretome, since it provides both coagulation-mediated platelet-derived factors, as well as hypoxia-induced signaling [37,38,39]. These secretomes therefore represent a developmental progression to platelet-based blood-derived products, such as platelet-rich plasma (PRP), which comprises exclusively platelet-derived growth factors and is currently accepted as the gold standard of blood-based regenerative therapies [45,46]. Instead of merely providing the initial (i.e., hemostasis-generated) phase of the wound-healing cascade, HPP and HPS supply angiogenesis-specific signaling that is naturally produced through hypoxia and, as a result, appear to be more angiogenic in vitro than PRP [39].

We have previously demonstrated that hypoxia preconditioned plasma (HPP), prepared using heparin anticoagulation, has approximately the same VEGF concentration as hypoxia preconditioned serum (HPS) but a much lower concentration of the platelet-derived angiogenesis inhibitors TSP-1 and PF-4 [39]. Here, we could confirm these findings in a larger sample of healthy subjects not using oral anticoagulation (Figure 3). Importantly, the current data verifies the positive effect of hypoxic conditioning on optimizing the proangiogenic composition of blood-derived secretomes, shown here by the significant increase in the HPS VEGF level compared to the normal serum (Figure 3). This difference translated into a more potent angiogenic response induced in endothelial cell cultures by HPP and HPS compared to the normal plasma and serum, respectively (Figure 5). These results suggest that the enrichment of blood-derived secretomes with hypoxia-induced protein factor signaling, beyond the level that can normally be obtained through platelet factor release, could enhance their angiogenic potency, thus making these therapies suitable for treating chronic wounds and ischemic tissues and possibly be more effective than solely platelet-based products such as PRP [39].

The primary question tested in this study was whether the long-term (six months) use of oral anticoagulation influences the angiogenic composition of hypoxia preconditioned secretomes. In our past work, we showed that the concentrations of the platelet-derived angiogenic inhibitors TSP-1 and PF-4 in both fresh plasma and HPP were significantly lower than that in HPS and platelet-rich plasma (PRP), indicating that the anticoagulation of blood prior to incubation effectively reduces platelet activation, even after static blood conditioning for four days [39]. Furthermore, the method of blood anticoagulation used for HPP preparation (EDTA vs. heparin) appeared to have an influence on the VEGF and TSP-1 protein levels, with lower concentrations observed in HPP obtained through EDTA compared to heparin anticoagulation but not PF-4 levels [39]. These previous findings provided a solid basis for developing our formal hypothesis, namely that long-term OA administration might alter the proteomic compositions of HPP and/or HPS. To examine this, the subjects were divided into three groups, representing three of the most widely used oral blood anticoagulants: (1) acetylsalicylic acid (ASA), (2) a combination of ASA + clopidogrel, and (3) nonvitamin K antagonist oral anticoagulants (NOACs) (Table 1A). The ELISA results presented here show that all three OA regimes significantly reduced the concentration of VEGF in HPP (compared to the control HPP), but not in HPS, to the extent that the HPP VEGF appeared significantly lower than that of HPS (something that was not observed either here or in previous studies when no OA was used [39]) (Figure 3). Furthermore, while clotting appeared macroscopically intact in all HPS samples obtained from subjects using OA (Figure 2), PF-4 levels in these samples were significantly lower than those in HPS obtained from subjects not using OA (Figure 3), suggesting a possible interference of OA administration with platelet activation in situ, and factor release. Nonetheless, given that VEGF, TSP-1, and PF-4 concentrations in HPS samples were significantly higher than in HPP samples obtained with all three OA regimes, it is safe to infer that OA administration does not completely abolish platelet activation during blood conditioning (provided that the extra amount of protein in HPS was platelet-derived). On the other hand, when considered in combination, the observed reductions in the HPP VEGF level and HPS PF-4 level suggest that OA use might influence both the PBC-generated hypoxia-induced signaling phase, as well as the platelet-derived factor release.

With respect to the angiogenicity of hypoxia preconditioned secretomes, we found that OA administration did not abolish the ability of HPP and HPS to stimulate microvessel formations in vitro (Figure 5). Indeed, HPP and HPS samples prepared from subjects using any of the three OA regimes tested could induce a complex capillary-like network in endothelial cell cultures following 12-h incubation. We did observe, however, that OA administration had a weakening effect (significant in samples obtained from ASA- and NOAC-treated subjects) on the angiogenic potential of HPP, which appeared reduced by half in comparison to HPP samples obtained without OA use (Figure 5), suggesting that the observed reduction in the OA-associated HPP VEGF level (Figure 3) might be responsible for this change. This finding may not be surprising, after all, given that the HPP concentrations of the angiogenic inhibitors TSP-1 and PF-4 were comparable with and without OA use (Figure 3), potentially making room for the concentrations of proangiogenic factors, such as VEGF, to assume a more determining role on the secretome’s net angiogenic effect. These results provide useful insight into the clinical utility of hypoxia preconditioned blood-derived secretomes in patients receiving OA, since they suggest that HPS may be a better alternative to HPP when choosing a tool to stimulate/support tissue angiogenesis and perfusion.

A large number of studies have already been published on the aforementioned OAs. ASA, a classical NSAID, has been used in broad conditions, including fever, pain, and inflammatory disease [47]. ASA inhibits cyclooxygenase-1 (COX-1) in platelets, thereby blocking the production of prostaglandins [48] and the production of thromboxan-A_2_ (TXA_2_), leading to reduced platelet activation and aggregation [49]. Previous studies have demonstrated that ASA and its derivatives can alter the VEGF expression, leading to suppressed angiogenesis as a consequence, although how ASA decreases the VEGF expression remains unknown [50,51,52,53]. For example, in hypertensive patients who had significantly higher plasma levels of VEGF, there were significant reductions in VEGF plasma concentrations while following the treatment with ASA for three months, indicating that the use of ASA leads to a reduction in intraplatelet angiogenic growth factors and platelet activation [54]. ASA has also previously been shown to decrease the plasma levels of VEGF in patients with ischemic heart disease who were undergoing coronary artery bypass grafting [55]. Furthermore, a daily use of low-dose ASA has been reported to reduce VEGF, platelet-derived growth factor (PDGF)-AB, and transforming growth factor beta-1 (TGF-beta1) expressions in freshly isolated human PRP [56]. These changes, also encountered in our data, can be attributed to alterations in the platelet activation and degranulation-mediated release of stored growth factors, e.g., VEGF, PDGF, EGF, insulin-like growth factor-1(IGF-1), basic fibroblast growth factor (bFGF), TGF-beta1, TSP-1, and PF-4 [57,58,59,60,61]. ASA might affect other blood cell types, as well. For example, ASA was found to block the differentiation of macrophages from bone marrow cells in vitro and decrease cell numbers, phagocytosis, and immunogenicity of mouse macrophages in vivo [62]. ASA was also shown to inhibit the tissue recruitment of monocytes/macrophages by impeding their adhesion process through COX-independent mechanisms [63]. Moreover, ASA may exert significant anti-inflammatory effects by suppressing the production of macrophage-derived inflammatory mediators [64]. As suggested by our results, such alterations in the PBC behaviors and growth factor expression affect the proteomic composition of PBC-derived secretomes, which further influences their angiogenic activity. Beyond that, the presence of ASA in the blood might also have direct effects on the endothelial cell function. Indeed, NSAIDs have been shown to inhibit angiogenesis in rodent models [47,65], while ASA significantly blocks the in vitro migration and capillary-like structure formation by endothelial cells [66]. It is therefore likely that the observed effects of ASA administration on the HPP angiogenic function might stem from a combination of reduced proangiogenic factor levels, as well as the direct inhibition of endothelial cells. Further studies are required before the relative significance of these mechanisms can be clarified.

A typical clinical combination of oral anticoagulants is that of ASA and clopidogrel. Clopidogrel targets the ADP P2Y12 receptor on platelets, responsible for irreversible platelet aggregation in response to ADP [67]. It has been shown that a seven-day intake of clopidogrel does not significantly modify the plasma concentrations of angiogenic factors VEGF-A, placenta growth factor, and stromal cell-derived factor-1 or biomarkers of endothelial cell activation [67]. On the other hand, clopidogrel inhibits the angiogenesis of gastric ulcer healing at least partially through inhibition of the VEGF-VEGFR2-ERK signal transduction pathway and, also, inhibits VEGF-stimulated HUVEC proliferation via the downregulation of VEGFR-2 and pERK [68]. Here, we did not include a clopidogrel-only group, as it is clinically rare, which makes it difficult to examine the isolated effect of clopidogrel on HPP/HPS angiogenic functions. Our results indicate that a combination of ASA and clopidogrel does not significantly alter the secretomes’ angiogenic composition (with respect to the three protein factors tested) compared to the ASA-only treatment, although the angiogenic response produced was somewhat stronger, effectively preventing the significant reduction in tube formation relative to the control HPP/HPS samples induced by ASA-only administration (Figure 5B). This is particularly interesting, since the daily ASA dose (100mg) in the combination group was the same as with the ASA-only group. Whether this effect is due to an interaction between ASA and clopidogrel or a direct effect of clopidogrel needs to be further examined. 

Following the launch of nonvitamin K antagonist oral anticoagulants (NOACs), taking Warfarin (vitamin-K antagonist) became rather rare, so that a sufficient number of Warfarin-treated subjects could not be recruited for this study. Over the past 50 years, Warfarin has been used as the mainstream drug for the prevention of stroke and systemic thrombo-embolism, before being surpassed by NOACs due to some clinical benefits (e.g., fewer drug interactions) [69,70]. NOACs, such as Rivaroxaban and Apixaban, are factor Xa inhibitors that block the generation of thrombin [71]. Factor Xa occupies a central position within the coagulation cascade as a convergence point between the intrinsic and extrinsic pathways and might play a key role in the regulation of angiogenesis [72]. Thrombin is known to inhibit the growth and branching of vascular tubules in vitro [39,72,73], while recombinant FX/FXa inhibits angiogenesis in HUVEC cultures in vitro [72] and was also found to be antiangiogenic when tested in in vivo models such as the zebrafish intersegmental vasculature (ISV) formation assay and the chick embryo chorioallantoic membrane (CAM) assay [72]. Furthermore, it has been demonstrated that the antiangiogenic effect of FXa is mediated through protease-activated receptor-1 (PAR-1) [72], which, in itself, has been reported to be both pro- and antiangiogenic; PAR-1 activation, when accompanied by low levels of thrombin, enhances angiogenesis in HUVEC cultures [72,74], while high concentrations of thrombin inhibit angiogenesis [72,75]. Our results indicate that HPP and HPS obtained from NOAC-treated subjects had a similar proteomic composition and angiogenic activity in the tube formation assay as HPP/HPS prepared from ASA-treated subjects (Figure 3 and Figure 5). As with the other two OA groups tested, the NOAC treatment appeared, on one hand, to reduce HPP angiogenicit;, on the other, it did not significantly reduce the angiogenic effectiveness of HPS, which remained more potent than that of the normal serum. It is possible that any FXa/thrombin-mediated effects of the NOAC treatment on cultured endothelial cells, that might well be angiogenesis-supporting [72], are counterbalanced by the reduction in platelet activation and proangiogenic factor release in HPP/HPS, although this requires further investigation. 

In this study, the presence of diabetes mellitus (DM) in the OA-treated subject population (7 out of the 25 subjects tested) was identified as a potential bias for the observed results, since DM is known to affect both leukocyte and platelet functions [76,77]. Indeed, chronic and acute hyperglycemia can trigger platelet activation [78,79], while, in diabetic patients, the production of several growth factors involved in initiating and sustaining the healing process are compromised [18]; for example, VEGF and TGF-beta protein expressions are reduced in diabetic dermal wounds [80,81]. Here, we examined the effects of DM independently of OA administration. We found no significant differences in either the proteomic composition or angiogenic activity of HPP and HPS obtained from diabetic subjects, compared to nondiabetic controls (Figure 3 and Figure 5). The type of diabetes (type 1 vs. type 2) did not appear to have an effect, either. These findings suggest that the application of hypoxia preconditioning as a tool for improving the angiogenic potency of blood-derived secretomes may be able to bypass aberrances in angiogenic functions that are a direct consequence of the disease state of diabetes.

A major limitation of this study is that, compared to the healthy, young subjects not using OA, OA-treated subjects were older and suffered from cardiovascular disease (Table 1A). Furthermore, none of the healthy subjects smoked, while one in five OA-treated subjects was a smoker. It is indeed known, by virtue of clinical observations, that an advanced age affects wound healing and is accompanied by the impairment of angiogenesis [82,83,84], while various wound-healing cellular processes demonstrate characteristic age-related changes [85]. For example, leukocytes display an age-related increase in the secretion of many inflammatory mediators [85,86,87,88], while the infiltration of macrophages and B-lymphocytes into wounds is delayed in models of wound healing in middle-aged and elderly mice [85,89]. The production of growth factors by macrophages declines with age as well [85,90]. As for the effect of smoking, several angiogenic factors have been found to be enhanced by nicotine [91]. For instance, nicotine induces the expression of proangiogenic growth factors bFGF, PDGF, and VEGF in endothelial cells [91,92,93,94,95]. In our study, nicotine in the plasma/serum of smokers might have also directly influenced the angiogenic response, since it is known that nicotine stimulates proliferation and tube formation by endothelial cells in vitro [94]. It is thus not possible to say with certainty that the observed differences in the secretome proteomic composition and angiogenic activity were the result of OA use or a consequence of other factors (e.g., old age, comorbidities, and smoking). Further studies, using larger patient samples (e.g., >20–30 subjects per group, based on similar studies), are therefore required before the effects of such confounding factors can be individually assessed, so that the findings of this study can be clearly validated. Nonetheless, based on the data presented in this work, it is reasonably safe to argue that OA administration, even in older patients who smoke, does not inhibit the angiogenic activity of hypoxia-preconditioned blood-derived secretomes, and therefore, it does not prohibit their potential application as tools for promoting therapeutic angiogenesis.

## 5. Conclusions

The findings of this study highlight the fact that neither OA administration nor DM (T1D or T2D) appear to inhibit the ability of HPP or HPS to induce microvessel formations in vitro, despite the significantly reduced VEGF concentrations in HPP and PF-4 levels in HPS, compared to non-OA controls. These findings also, once again, confirm the positive effect of hypoxic conditioning on optimizing the angiogenic potential of blood-derived secretomes and suggests that OA use does not prohibit the application of these products for supporting tissue vascularization and wound healing. Therefore, patients requiring angiogenesis-promoting therapies for central/peripheral ischemia and chronic wounds could potentially still benefit from these new-generation autologous and bioactive treatments.

## Figures and Tables

**Table 1:** Demographic data.

**A**	**No OA**	**ASA**	**ASA + Clopidogrel**	**NOACs**
**Total number**	5	8	10	7
Male/Female	4/1	6/2	9/1	5/2
**Mean age ± SD** (years)	26.0 ± 3.8	71.8 ± 5.9	71.5 ± 6.4	67.0 ± 8.6
**moking** (number of subjects)	0	1	4	0
**Diabetes Mellitus** (number of subjects)	0	4	2	1
**B**	**No DM(No OA)**	**T1D(No OA)**	**T2D(No OA)**	
**Total number**	8	6	10	
Male/Female	4/4	4/2	1/9	
**Mean age ± SD** (years)	30.75 ± 3.99	39.0 ± 5.88	62.4 ± 18.55	
**BMI** (kg/m²)	20.87 ± 2.50	26.34 ± 4.92	40.7 ± 15.16	
**Blood glucoseat the time of blood collection** (mg/dL)	98.75 ± 5.58	126.84 ± 37.74	122.2 ± 31.58	

Patient demographics of different subject groups included in (**A**) oral anticoagulation (OA) experiments to assess the effects of three of the most widely used oral blood anticoagulants: group 1, acetylsalicylic acid (ASA) (100 mg/day), group 2, a combination of ASA + clopidogrel (Plavix) (100 mg + 75 mg/day), and group 3, nonvitamin K antagonist oral anticoagulants (NOACs) (e.g., Apixaban and Rivaroxaban) (dose was dependent on the pathology and drug type). All subjects were under a consistent blood anticoagulation regime in the past 6 months and (**B**) diabetes mellitus (DM) experiments, further differentiated into type 1 (T1D) and type 2 (T2D) diabetes, in order to assess the effect of DM independently of OA use. Subjects had a clinical diagnosis of DM for longer than one year prior to initiation of the study. BMI = body mass index and SD = standard deviation.

## Abbreviations

ASA	Acetylsalicylic acid
bFGF	basic fibroblast growth factor
COX1	Cyclooxygenase-1
EGF	epidermal growth factor
EWS	Extracorporeal Wound Simulation
HPP	hypoxia preconditioned plasma
HPS	hypoxia preconditioned serum
HUVECs	human umbilical vein endothelial cells
IGF-1	insulin-like growth factor-1
NOACs	Nonvitamin K antagonist oral anticoagulants
OA	oral anticoagulation
PAR1	Protease-activated receptor-1
PBC	peripheral blood cells
PBS	phosphate buffered saline
PDGF	platelet-derived growth factor
PF-4	platelet factor-4
TGF-beta1	transforming growth factor beta-1
TSP-1	thrombospondin-1
TXA2	thromboxan-A_2_
VEGF	vascular endothelial growth factor
